# A systematic approach for identifying shared mechanisms in epilepsy and its comorbidities

**DOI:** 10.1093/database/bay050

**Published:** 2018-06-03

**Authors:** Charles Tapley Hoyt, Daniel Domingo-Fernández, Nora Balzer, Anka Güldenpfennig, Martin Hofmann-Apitius

**Affiliations:** 1 Department of Bioinformatics, Fraunhofer Institute for Algorithms and Scientific Computing, Konrad-Adenauer-Straße, Schloss Birlinghoven, 53754 Sankt Augustin, Germany; 2 Department of Life Science Informatics, Bonn-Aachen International Center for IT, Rheinische Friedrich-Wilhelms-Universität Bonn, Endenicher Allee 19C, Bonn 53113, Germany

## Abstract

Cross-sectional epidemiological studies have shown that the incidence of several nervous system diseases is more frequent in epilepsy patients than in the general population. Some comorbidities [e.g. Alzheimer’s disease (AD) and Parkinson’s disease] are also risk factors for the development of seizures; suggesting they may share pathophysiological mechanisms with epilepsy. A literature-based approach was used to identify gene overlap between epilepsy and its comorbidities as a proxy for a shared genetic basis for disease, or genetic pleiotropy, as a first effort to identify shared mechanisms. While the results identified neurological disorders as the group of diseases with the highest gene overlap, this analysis was insufficient for identifying putative common mechanisms shared across epilepsy and its comorbidities. This motivated the use of a dedicated literature mining and knowledge assembly approach in which a cause-and-effect model of epilepsy was captured with Biological Expression Language. After enriching the knowledge assembly with information surrounding epilepsy, its risk factors, its comorbidities, and anti-epileptic drugs, a novel comparative mechanism enrichment approach was used to propose several downstream effectors (including the GABA receptor, GABAergic pathways, etc.) that could explain the therapeutic effects carbamazepine in both the contexts of epilepsy and AD. We have made the Epilepsy Knowledge Assembly available at https://www.scai.fraunhofer.de/content/dam/scai/de/downloads/bioinformatik/epilepsy.bel and queryable through NeuroMMSig at http://neurommsig.scai.fraunhofer.de. The source code used for analysis and tutorials for reproduction are available on GitHub at https://github.com/cthoyt/epicom.

## Introduction

Seizures are transient occurrences of signs and symptoms due to abnormal or excessive neuronal activity in the brain (1). Classically, their underlying causes were thought to be the primary drivers for increasing mortality in epilepsy. While epilepsy has been classically studied as a disorder of the brain characterized by an enduring pre-disposition to epileptic seizures, it is no longer considered a condition in which seizures are the only concern (2). Epilepsy is also associated with several comorbidities, including Alzheimer’s disease (AD), Parkinson’s disease (PD), other nervous system diseases and psychiatric disorders (3–5), due to a variety of genetic, biological and environmental factors (6).

The prevalence of migraine in epilepsy patients (under 64 years old) is 5.71% in contrast to 3.47% in the general population (7). The mechanistic understanding of epilepsy and migraine presumes that they share the underlying pathophysiology related to alterations in sodium and calcium ion channels and ion transporters (sodium-potassium transport; 8, 9). Moreover, drugs acting on voltage-gated sodium channels and γ-aminobutyric acid (GABA) receptors (e.g. valproate, topiramate, etc.) are used not only prevent migraine attacks, but are also used as anti-epileptic drugs (10, 11).

The prevalence of epileptic seizures in AD patients is strongly influenced by genetic factors (12)—epilepsy and seizures occur more often in patients with early-onset familial AD than those with sporadic AD (13). Further, convulsive seizures have been described in approximately 40% of familial AD patients with the PSEN1 p.Glu280Ala mutation (14), 30% with PSEN2 mutations (15) and 57% with amyloid precursor protein (APP p.Thr174Ile) duplications (16). Additionally, the p.Pro86Lys mutation in CALHM1 (rs11191692) is associated with both AD and temporal lobe epilepsy through its influence on calcium homeostasis (17).

Evidence that some comorbidities (e.g. AD and depression) also act as risk factors for developing seizures suggests they may share pathophysiological mechanisms (13, 18). Conversely, epileptic seizures (as well as neoplasms) have been reported to cause intellectual disabilities in patients with tuberous sclerosis (19, 20). Furthermore, certain anti-epileptic drugs (e.g. topiramate) are associated with higher incidence of cognitive problems (1, 21).

The several causal and associative relationships observed between epilepsy and other indications on the phenotypic level warrant further investigation for shared elements on the genetic and molecular levels. While inquiry on the genetic level often begins with genome-wide association studies to identify shared loci, identifying the appropriate data set(s) and linking intergenic single nucleotide polymorphisms (SNP) to their functional consequences across scales in complex disease is still a significant challenge (22). Even after identifying disease-associated genes, it is difficult to assess their individual contributions to the complex etiology of epilepsy and its related indications. Thus, capturing the different causal relationships between biological entities involved in the pathophysiology of a complex disease is an essential step to a better understanding of the processes that lead to the disease state.

**Figure 1.**
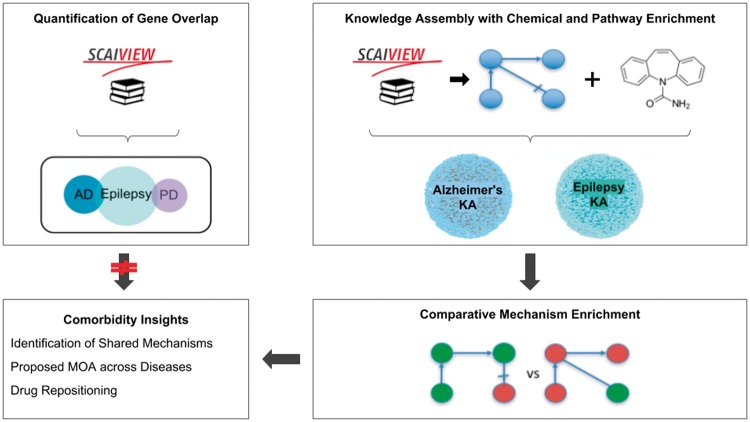
A graphical abstract of the methodology, analysis, and results presented in this work. The two upper boxes represent the methodology while the two lower boxes represent the analysis and results. The upper-left box outlines the quantification of gene overlap between epilepsy and its well-known comorbidities described in Keezer *et al.* (23; e.g. AD, PD, etc.) using literature based methods. The upper-right box outlines the assembly of knowledge from epilepsy literature with chemical and pathway enrichment as described in the ‘Preparation for Mechanism Enrichment’ and ‘Relation Extraction’ Sections. The lower-right box represents the comparative mechanism enrichment that was used to generate comorbidity insights (lower-left box) after literature-based methods proved insufficient.

Here, we present two methods to hypothesize shared mechanisms: first, a literature-based approach for quantifying gene overlap between epilepsy and its comorbidities as a proxy for a shared genetic basis of disease, or genetic pleiotropy; and second, a systematic approach using the NeuroMMSig mechanism enrichment server. Finally, we use these methods to propose an explanation for the observed therapeutic effects of a drug that has been studied in the contexts of epilepsy and AD. A schematic representation of the methodology, analysis and results is presented in Figure 1.

## Materials and methods

### Pre-processing of epidemiological studies

Epidemiological studies comparing the incidence of several comorbidities of epilepsy versus incidence in the general population were extracted from a recent review by Keezer *et al.* (23). The prevalence ratio, which describes the ratio of incidence of a given condition in epilepsy patients versus the general population, was calculated for each study.

### Quantification of gene overlap

SCAIView v1.7.3, indexed from MEDLINE on 2016-07-14, (http://academia.scaiview.com/academia) was used to identify and quantify the overlap of genes co-occurring in the literature with epilepsy and its novel and well-known comorbidities reviewed by Keezer *et al.* Three comorbidities were excluded from assessment by literature-based methods due to their poor correspondence with MeSH terms (i.e. allergie) or their lack of specificity (i.e. heart disease and neoplasms) by occurring too frequently and covering too many genes to be insightful. Finally, additional epidemiological comorbidity studies with PD were curated and included due to its previously published relevance (24).

In order to later assess publication bias in literature-based methods, the total number of documents associated with each disease was reported by querying SCAIView with each disease’s corresponding MeSH term. The total number of associated genes with each disease was counted by those with a positive relative entropy (i.e. occur more frequently in the results of a given SCAIView query than in the rest of the SCAIView indexed literature) in the context of the query as described by Younesi *et al.* (25). Gene sets for each comorbidity were then retrieved by constructing queries using their corresponding MeSH terms joined with the epilepsy MeSH term by the ‘AND’ operator. The associated genes for each comorbidity query were identified with the same method and the epilepsy pleiotropy rate was calculated as the percentage of the genes associated with the comorbidity query in the set of associated genes with epilepsy (Table 1).

**Table 1.** Results of the Epilepsy comorbidity analysis using SCAIView

Disease (MeSH ID)	Associated documents	Disease associated genes	Comorbidity associated genes	Epilepsy pleiotropy rate (%)
Epilepsy (D004827)	192 245	2901	––	––
Stroke (D020521)	210 846	4533	633	17.78
AD (D000544)	109 495	4968	396	13.65
Migraine (D008881)	30 928	1230	306	10.54
PD (D010300)	79 103	3646	258	8.89
Hypertension (D006973)	391 190	5574	252	8.68
Dementia (D003704)	183 802	5833	220	7.58
Diabetes mellitus (D003920)	394 411	6661	184	6.34
Intestinal diseases (D007410)	629 691	9093	166	5.72
Thyroid diseases (D013959)	153 025	4366	133	4.58
Anxiety (D001007)	84 138	1782	124	4.27
Arthritis (D001168)	259 327	5367	122	4.2
Cataract (D002386)	52 150	2238	119	4.1
Asthma (D001249)	147 697	3761	86	2.96
Glaucoma (D005901)	56 679	2303	48	1.65
Depressive disorder, major (D003865)	15 706	1249	46	1.58
Urinary incontinence (D014549)	34 170	720	24	0.82
Peptic ulcer (D010437)	68 234	1445	21	0.72
Back pain (D001416)	48 516	1191	17	0.58
Pulmonary disease, chronic obstructive (D029424)	35 627	2244	15	0.51
Fibromyalgia (D005356)	9021	468	10	0.34
Emphysema (D004646)	25 511	1261	9	0.31
Bronchitis, chronic (D029481)	9085	580	2	0.06

Notes: The number of ***associated documents***(column 2) retrieved from SCAIView for each disease is shown given a reference query using corresponding the MeSH term from column 1. The ***disease-associated genes***(column 3) contain the number of genes relevant to the corpus retrieved from a disease-specific query. The ***comorbidity-associated genes***(column 4) contain the number of genes relevant to the comorbidity query between the target disease and epilepsy. Lastly, the ***epilepsy pleiotropy rate***(column 5) describes the ratio of the count of genes reported in column 4 with the total number of epilepsy-associated genes (2901).

For example, 226 genes were found to be associated with diabetes using the comorbidity query [MeSH Disease:”Epilepsy”] AND [MeSH Disease:”Diabetes Mellitus”]. Of these, 184 had positive relative entropies, which indicate that these genes occur more frequently in literature mentioning both diseases than in the rest of the SCAIView indexed literature. Finally, the epilepsy pleiotropy rate was calculated normalizing 184 by the total number of genes (2901) with a positive relative entropy found by querying for epilepsy [MeSH Disease:”Epilepsy”], resulting in an epilepsy pleiotropy rate of 6.34%.

### Relation extraction

While literature co-occurrence can generate initial hypotheses about genetic pleiotropy, it does not provide sufficient mechanistic insight to explain the clinical observations in epilepsy and its comorbidities. The increasing quantity of knowledge in the biomedical domain makes it difficult or impossible for researchers to be knowledgeable in any but incredibly specific topics (26). The task of manually generating pleiotropy hypotheses that explain overlap between the aetiological mechanisms of epilepsy and its comorbidities is daunting. In order to enable computer-aided automatic reasoning, the knowledge surrounding epilepsy and its comorbidities was systematically extracted from the literature using manual relation extraction and encoded in Biological Expression Language (BEL; 27).

First, a corpus was generated from the 192 245 documents related to epilepsy retrieved (Table 1) to further investigate the causal relations surrounding the identified genes. A second corpus was generated from the 2666 documents retrieved by querying SCAIView for ‘epilepsy’ and its sub-terms in the Epilepsy Ontology (28) occuring with the free text, ‘comorbidity’. Manual relation extraction and encoding in BEL was then performed starting with a select subset of the two corpora based on their prioritization by SCAIView to generate the Epilepsy Knowledge Assembly. The knowledge assembly was further enriched with pharmacological knowledge surrounding 19 anti-epileptic drugs and their targets from the Pharmacogenetics and Pharmacogenomics Knowledge Base (PharmGKB; 29). Ultimately, the knowledge assembly comprised relations from 641 unique citations. Finally, the PyBEL framework (30) was used to parse and validate the syntax and semantics of the underlying BEL Script. A summary of the contents of the Epilepsy Knowledge Assembly is presented in Table 2.

**Table 2.** Statistics over the Epilepsy Knowledge Assembly generated by PyBEL, grouped by sub-graph

Sub-graph name	Biological entities	Relationships	Connected Components	Citations
Adaptive immune system sub-graph	12	12	4	5
Adenosine signaling sub-graph	76	154	3	15
Apoptosis signaling sub-graph	228	503	5	115
Brain-derived neurotrophic factor signaling sub-graph	75	142	1	29
Calcium dependent sub-graph	302	793	8	73
Chromatin organization sub-graph	8	10	2	2
Energy metabolic sub-graph	91	177	4	24
Estradiol metabolism	7	8	2	1
G-protein-mediated signaling	78	140	5	25
Gaba sub-graph	262	632	2	56
Glutamatergic sub-graph	121	246	5	32
Hormone signaling sub-graph	126	256	9	16
Inflammatory response sub-graph	42	49	4	21
Innate immune system sub-graph	41	63	8	18
Interleukin signaling sub-graph	46	111	3	17
Long term synaptic depression	64	129	2	21
Long term synaptic potentiation	125	252	2	46
Mapk-erk sub-graph	313	706	5	68
Metabolism	340	600	14	126
Mirna sub-graph	5	4	1	3
Mossy fiber sub-graph	38	66	2	14
Mtor signaling sub-graph	166	336	3	42
Neurotransmitter release sub-graph	552	1667	5	131
Notch signaling sub-graph	105	205	3	20
Protein kinase signaling sub-graph	377	850	6	87
Protein metabolism	129	185	8	44
Reelin signaling sub-graph	117	253	2	21
Regulation of actin cytoskeleton sub-graph	5	3	2	2
Serotonergic sub-graph	148	478	2	18
Thyroid hormone signaling sub-graph	106	228	2	9
Transport related sub-graph	49	60	7	25
Wnt signaling sub-graph	27	38	3	15
*Total*	*3478*	*12481*	*16*	*641*

Notes: The first column, ***biological entities*, **corresponds to the number of genes, chemicals, proteins, biological process, etc. in each sub-graph. The second column, ***relationships***(i.e. edges), corresponds to the number of connections between each sub-graphs’ biological entities. The third column, ***connected components*, **corresponds to the number of ‘connected’ groups of nodes within each sub-graph. The final column, ***citations*, **corresponds to the total number of articles from which information was extracted to build each sub-graph. A more detailed summary is included in the Supplementary Material.

### Preparation for mechanism enrichment

The Epilepsy Knowledge Assembly was enriched with mechanistic annotations following the procedures outlined by Domingo-Fernández *et al.* (24) in order to integrate it into NeuroMMSig and enable multi-modal mechanism enrichment analyses with queries over genes, SNPs and neuroimaging features.

A taxonomy of epilepsy mechanisms was generated by combining the list of well-established epilepsy mechanisms from Staley (31) with concepts from the Pathway Terminology System (32) co-occurring in articles matching either [MeSH Disease: ‘Epilepsy’] or entries in the Epilepsy Ontology indexed by SCAIView. The resulting 784 terms representing mechanisms were curated in order to normalize entities, remove irrelevant entries and group similar terms. Next, relations in the Epilepsy Knowledge Assembly were annotated with mechanisms based on whether their entities were involved in the mechanism as outlined by Domingo-Fernández *et al.* (24). During the annotation process, new mechanisms not yet included in the inventory were found in the literature; thus, the mechanism inventory was updated in parallel until concluding the annotation with a total of 32 annotated sub-graphs (Table 2). More details and examples about the mapping procedure can be found on the NeuroMMSig introduction page.

In the next section, NeuroMMSig is used to identify shared mechanisms between epilepsy and AD on the basis of the mechanism of action of multi-indication drugs.

## Results and discussions

### Investigation of comorbidities

Of the 2901 genes identified as associated with epilepsy by having a positive relative entropy score, Table 1 shows that nervous system conditions were among the highest literature-based gene overlap (10.54% of epilepsy genes co-occurred with migraine, 7.58% with dementia, 8.89% with PD and 13.65% with AD). While these conditions were also highly ranked in epidemiological studies by their prevalence ratios, literature-based gene overlap does not correlate with prevalence ratios across all of the conditions reported by Keezer *et al.* (Supplementary Figure S2) and therefore is the best tool for gaining insight into the comorbidities of epilepsy.

While literature-based methods may be a poor proxy for genetic pleiotropy and are generally insufficient for unraveling the shared pathophysiology in epilepsy and studied comorbidities, systematic approaches for identifying and evaluating shared mechanisms may better explain the aggregate effects of their interactions that cannot be captured by simple approaches like literature co-occurrence.

### Mechanism enrichment

AD was chosen as a putative comorbidity of epilepsy for further investigation not only because it had the highest literature-based gene overlap with epilepsy (Table 1), but additionally because of the prior existence of the AD Knowledge Assembly (33) and its inclusion in NeuroMMSig.

Of the most frequently mentioned drugs in epilepsy literature (Supplementary Figure S1), we identified carbamazepine as having both notable target representation in the AD Knowledge Assembly along with multiple references indicating its positive effects on memory and treatment of elderly patients with seizures (34) as well as positive effects in the treatment of AD patients (34, 35).

In the following sections, NeuroMMSig is first used to investigate the mechanisms enriched by the targets of carbamazepine in the context of epilepsy. After, a comparative enrichment is made to identify possible overlapping mechanisms with AD in order to explore the therapeutic effects of the drug in both disease contexts.

### Epilepsy mechanism enrichment

While carbamazepine has been observed to act through inhibition of sodium and calcium voltage-gated channels as well as activation of the GABA receptors (36, 37), its mechanism of action is still not fully understood (38). In order to better understand the impact of the drug, the gene set of all of its known targets (Supplementary Text S1), was queried on the NeuroMMSig mechanism enrichment server against the Epilepsy Knowledge Assembly.

Because the NeuroMMSig mechanism enrichment algorithm only provides relative scores, the top 10th percentile of results were used. Of the 12 networks with at least one mapped gene, those with an enrichment score in the top 10th percentile (above 0.696) were the adenosine signaling sub-graph and the GABA sub-graph (Supplementary Table S1). While the increase of the purine nucleoside, adenosine, has been associated with the incidence of seizures, its mechanistic connection is still unknown (39). However, recent research has identified several promising targets that regulate and balance adenosine levels such as adenosine kinase (ADK) and its receptors (ADORA family; 39, 40). Similarly to adenosine, the inhibitory neurotransmitter response induced by GABA is responsible for balancing many excitatory signals occurring in the brain. Studies that investigated reduction and abnormalities in GABA-inhibitory processes lead to the development of GABA agonists (e.g. vigabatrin and tiagabine) that act as anti-convulsants in epilepsy patients (41).

**Figure 2.**
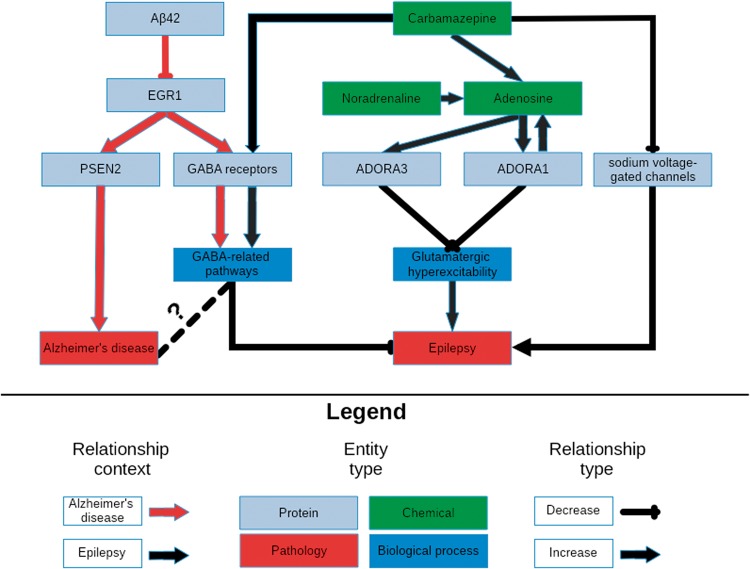
A schematic representation of the knowledge surrounding carbamazepine retrieved by querying its targets with NeuroMMSig. The relevant portions of the most significantly enriched graphs in the context of epilepsy and AD, the adenosine signaling and the GABA sub-graphs, were merged and displayed in order to highlight a potential explanatory mechanism for the therapeutic effects of carbamazepine (Supplementary Text S2). It is rendered with a hierarchical layout to mirror the flow from molecular entities to proteins, biological processes and pathologies.

After assessing the plausibility of these sub-graphs’ involvement in the aetiology of epilepsy, the union of the networks was used to further investigate the relation between the downstream effects of carbamazepine and its therapeutic effect on epilepsy patients. Due to the its size, it was necessary to filter and query the network by finding the shortest paths between the drug’s targets and different biological processes of interest in the context of epilepsy then combining them to form a new graph to support quickly identifying candidate pathways. Finally, the common upstream controllers between each pathway were included to provide further context to their overlap. Combining, reasoning over, and manually interpreting the generated paths lead to the simplified network depicted in Figure 2 representing the downstream effects of carbamazepine.

The figure demonstrates the synergistic effects of the activation of the GABA receptor family and the inhibition of the sodium voltage-gated channels to potentiate GABA-mediated inhibition, and therefore, decrease the risk of developing seizures. Furthermore, carbamazepine causes an increase in the production of adenosine, whose receptors, ADORA1 and ADORA3, in a positive feedback loop with the production of adenosine which ultimately leads to a reduction in glutamatergic excitatory signals. Synaptic plasticity in response to the downstream effects of these signals may contribute to drug resistance, and ultimately, seizure recurrence.

### Comparative mechanism enrichment

NeuroMMSig was queried with the same gene set in the context of AD in order to perform a comparative investigation of shared mechanistic perturbations with epilepsy. Because the mechanism of action of carbamazepine is poorly represented in the literature (Supplementary Table S2) and its known targets are less implicated in AD, it was unsurprising that fewer sub-graphs were enriched in the context of AD.

The most significant, the GABA sub-graph, which describes the upstream controllers of the GABA receptor and its downstream effectors, highly overlapped with the GABA sub-graph in the context of the epilepsy–it contains key relations that may explain the efficacy of carbamazepine in both conditions. Studies in AD models have shown a negative correlation between the abundance of amyloid beta 42 and the expression of the transcription factor for the GABA receptor family EGR1 (42, 43). This correlation could be caused by an unknown controller in the aetiology of AD; in which state, a patient would have decreased expression of EGR1 and therefore fewer GABA receptors and less ability to inhibit the excitatory signals that lead to seizures. While the link between epilepsy and GABAergic neurotransmission has already been exploited by anti-epileptic drugs, its role in AD is not yet clearly understood (Figure 2). However, several recent publications have rationalized targeting GABAergic neurotransmission for treatment of AD (44, 45).

Tangentially, EGR1 upregulates the expression of PSEN2, a member of the catalytic sub-unit of the γ-secretase complex that regulates APP cleavage (46). Mutations in PSEN2 that have been both linked to amyloid beta accumulation (47) and seizures in AD patients (48) provide further evidence for the existence for a shared mechanism through which carbamazepine acts in the contexts of AD and epilepsy (Figure 2).

Noradrenaline is a known anti-convulsant (49) that is often lacking in patients in the early stages of AD due to an observed loss of noradrenergic neurons (50). Because carbamazepine has been observed to activate noradrenergic neurons (51), its anti-convulsant activity may be due to it indirectly increasing noradrenaline levels. Finally, noradrenaline potentiates the previously mentioned adenosine pathways (52).

While mechanism enrichment provides several insights, a cursory search of PubMed of ‘Carbamazepine’[nm] AND ‘Alzheimer disease’[MeSH Terms] suggested publications (53) that implicate autophagy in the therapeutic action of Carbamazepine. Further investigation showed Carbamazepine does not have a significant effect on expression of proteins in the mTOR-pathway (53) and that it is likely increasing autophagic flux through an mTOR-independent pathway. Mechanism enrichment analysis was unable to prioritize autophagy pathways due to some of the shortcomings of knowledge-based methods. For example, the AD NeuroMMSig sub-graph corresponding to autophagy pathways did not contain any of the targets of Carbamazepine listed by PharmGKB. This could be due to the choice of boundaries in sub-graph definition, or also due to the lack of annotation of autophagy-related targets in PharmGKB. Autophagy has been implicated in epilepsy, but the literature has not yet succinctly described the connection from the therapeutic to its target, pathway, and finally the pathology. Finally, because knowledge assemblies are inherently incomplete, this shows the complementary nature of the two approaches.

While the exact mechanism of action of carbamazepine remains elusive, this proposed mechanism enrichment approach was able to identify multiple pathways through which it could be acting in both the AD, epilepsy and shared context. Looking forward, this approach can be applied across a wide variety of chemical matter in the neurodegenerative disease space, as well as in other domains for which appropriately annotated knowledge assemblies exist, in order to support identification of drugs’ mechanisms of actions, drug repositioning opportunities and the development of new lead compounds.

## Conclusion

Our findings indicate that literature-based methods as a proxy for genetic pleiotropy generally do not correlate with the results from epidemiological studies of epilepsy and its comorbidities. Furthermore, strictly gene-centric methods lack the ability to elucidate mechanistic insight that a knowledge assembly can support.

After formalizing a representative sample of the knowledge surrounding epilepsy, its risk factors, its comorbidities and anti-epileptic drugs, we annotated mechanistic sub-graphs to include in the NeuroMMSig mechanism enrichment server. Finally, an enrichment approach focusing on the targets of carbamazepine proposed the several downstream effectors (including the GABA receptor, GABAergic pathways, etc.) that could explain its therapeutic effects in both the contexts of epilepsy and AD.

Future work will include applying this procedure to a wider variety of drugs and chemical matter across different diseases. Finally, we have made the Epilepsy Knowledge Assembly publicly available through NeuroMMSig (http://neurommsig.scai.fraunhofer.de) to facilitate further systems biology and chemoinformatics investigations of epilepsy.

## Supplementary data

Supplementary data are available at *Database* Online.

## Supplementary Material

Supplementary Data
